# Predictors of health-related quality of life in Serbian patients with head and neck cancer

**DOI:** 10.4317/medoral.25274

**Published:** 2022-04-03

**Authors:** Miloš Čanković, Milan Tešić, Marija Jevtić, Dejan Stevanović, Milan B Jovanović, Dejan Kostić, Jadranka Antić, Sanja Krejović Trivić

**Affiliations:** 1 University of Novi Sad, Faculty of Medicine; 2 Dentistry Department, Oral medicine section, Novi Sad, Serbia; 3 Military Medical Academy, Clinic for Maxillofacial surgery, Belgrade, Serbia; 4 Institute of Public Health of Vojvodina, Novi Sad, Serbia; 5 Clinic for neurology and psychiatry for children and youth, Belgrade, Serbia; 6 University of Belgrade, Faculty of Medicine; 7 Clinical Hospital Center Zemun, Department of ENT and Maxillofacial surgery, Belgrade, Serbia; 8 University of Defense, Faculty of Medicine of Military Medical Academy; 9 Military Medical Academy, Institute of Radiology, Belgrade, Serbia; 10 University Clinical Center of Serbia, Clinic for Endocrinology, Diabetes and Metabolic Disease, Department of Endocrine tumors, Belgrade, Serbia; 11 University Clinical Center of Serbia, Clinic for ENT and Maxillofacial surgery, Belgrade, Serbia

## Abstract

**Background:**

The aim of this study was to identify predictors of the Health-Related Quality of Life (HRQoL) in patients with head and neck cancers (HNCs).

**Material and Methods:**

In total, 345 patients with HNCs were interviewed. A self-report questionnaire was administered to collect data about demographic characteristics, health status, smoking, alcohol consumption habits, and HRQoL. It were used the EORTC Instruments - Quality of Life Questionnaire-Core 30-questions (QLQ-C30), Quality of Life Questionnaire - Head and Neck Module 35-questions (QLQ-H&N 35) and OHIP-14 instrument for HRQoL assessments. Clinical information and treatment data were collected from medical records.

**Results:**

Five groups of HRQoL predictors were identified: demographic, socioeconomic, behavioral, psychophysical, and clinical/treatment. These HRQoL predictors had a strong (i.e., age, level of social support and social contact, level of education, depression, fatigue, presence of gastrostomy, comorbidities, and use of pain medications and supplements), a moderate (i.e., marital status, smoking, sexuality problems, time since diagnosis, presence of tracheostomy, and side effects outcomes of radio and chemotherapy) and a small impact (i.e., employment/financial difficulties, tumor site and stage, and surgical procedure).

**Conclusions:**

Study identified nineteen predictors that had significant, moderate and small impact on the HRQoL of patients with HNCs. Some of the predictors, like levels of social support and social contact, depression, and comorbidities could be targets for innervations to improve HRQoL.

** Key words:**Quality of Life, oral health, combined modality therapy, treatment outcome.

## Introduction

The term head and neck cancer (HNC) refer to the primary tumors originating from the structures of the larynx, pharynx, oral cavity, paranasal cavities, and salivary glands (1). Tobacco and alcohol use are a leading cause of HNC in about 75% of cases, and in addition to that, human papillomavirus (HPV) is the cause in 70% of cases of oropharyngeal carcinoma (2).

The standard in the treatment of HNC is based on tumor site and TNM stage (2). Early stages (I/II) are treated with a single modality therapy - surgery or radiotherapy (RT), depending on the tumor site and its extent, expected cure rate, as well as functional outcome and cosmesis (physical disFigurement; (2). Patients in advanced stages (III/IV) are treated with multimodal therapy, which includes surgery, RT and chemotherapy (CHT; (2). Thus, as HNC affects the physical structures necessary for normal functions (speech, chewing, swallowing, breathing, etc.), and therapy can lead to the deformities that negatively reflect psychosocial functioning, it is of great interest to evaluate the biopsychosocial consequences of the HNC and its modality treatment (1), especially if viewed form experiential frame of patients' (3). To achieve this goal, clinical studies require a response to treatment, survivorship and HRQoL (1-3).

Therefore, studies found a large number of clinical, therapeutic and socio-behavioral factors that are important predictors of the HRQoL of these patients (1,4-8). Some of the predictors with the strongest negative impact were the presence of a feeding tube and comorbidities (4). Predictors with moderate negative impact were the time since diagnosis, tracheotomy, modality treatment with the RT and CHT, tumor site and stage (4-6,9), while demographic data - age, unemployment, marital and socioeconomic status (8,10), behavioral data - alcohol consumption and smoking (2), sexual habits (11), family problems and social support (12) and psychic distress and depression (13) proved to be significant indicators to consider when deciding on conducting a treatment procedure.

Clinical characteristics as significant predictors of functional status are tumor attribute - site and stage, and modality treatment - surgery, RT and CHT (5,6,9,14). Modality treatments combined together, may cause side effects that can be very limiting for the patients (9). This, observed in the physical status, can lead to the lower value of body mass index due to malnutrition and loss of muscle mass (15), and consequently has repercussions on performing basic and Instrumental Activities of Daily Living (ADLs/IADLs; (16).

A number of cross-sectional studies have evaluated the predictors of HRQoL in a sample of patients with primary cancer at specific site and/or treated with specific therapeutic modality (1,6). Very little studies (1,3,4,9), and none in Serbia, have been conducted on patients with different site of HNC treated with a combination of treatment modalities; all together, as part of a larger intervention. This study aimed to determine the association of demographic, socioeconomic, and behavioral risk factors, as well as the clinical characteristics of the disease and the therapeutic models of treatment with HRQoL in patents with HNC.

## Material and Methods

- Participants

This is a cross-sectional study, conducted at the ENT Department with Maxillofacial surgery of Clinical Hospital Centre Zemun, Belgrade, Serbia, in a period January the 1st 2014 - June the 30th 2018. The participation was on voluntary basis and to all participants was first explained the aim of the study, and then they signed provided written informed consent. The inclusion criteria were: patients with a histopathologically verified diagnose of squamous cell carcinoma of HN structures; upon completion and/or during the therapeutic procedure (surgical treatment or surgical treatment and/or RT and CHT); age, 18 years old or older.

Data for all analyses were available for 345 patients with HNC. The clinical characteristics of disease were collected from medical records: diagnosis (based on histopathological conformation), time since diagnosis, tumor site and stage (TNM classification), treatment modality (surgery, surgery with RT and/or CHT), presence of percutaneous gastrostomy (PEG) and tracheostomy (at the time of survey). Depending on the performed surgery, all patients were classified into two groups: those with non-mutilant (surgical excision i.e., extirpation of tumor without anatomical and functional deficits) and with mutilant (in addition to surgical excision i.e., extirpation, tissue resection and/or neck dissection was performed which resulted by anatomical and functional deficits). The modality treatment for each patient was decided by an oncological team within the standard treatment protocols. Basic and clinical characteristics of the patients are given in Table 1.

**Table 1:**
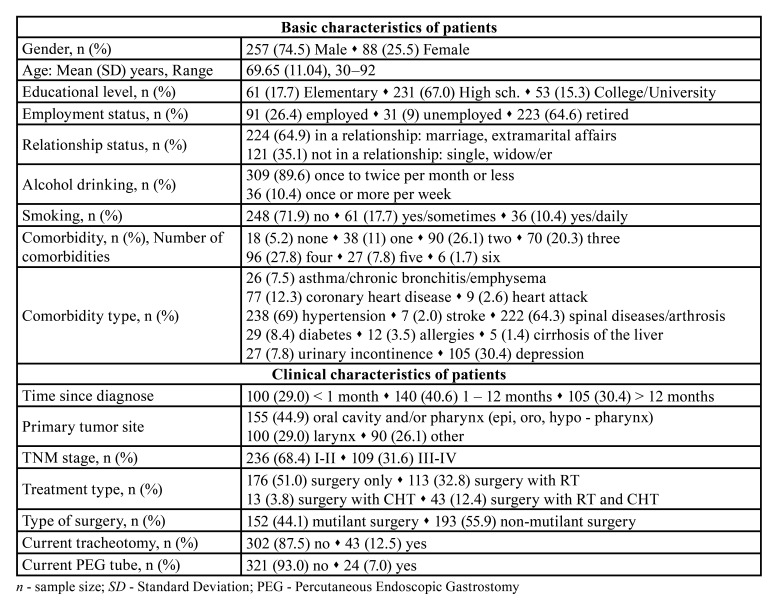
Basic and clinical characteristics of patients (n = 345).

- Instruments

- European Organization for Research and Treatment of Cancer (EORTC) QLQ-C30 and QLQ-H&N35.

The QLQ-C30 is a 30-item instrument for HRQoL assessments that covers the cancer specific symptoms, treatment side-effects, and functional problems for all cancer patients (17). The items are grouped into six functional scales and nine symptom scales. TheQLQ-H&N35 is a 35-item instrument for the assessment of symptoms associated specifically with HNC and its questions are grouped into seven subscales (18). For both instruments the answers were converted into linear scoring scale from 0 to 100, where scores of 100 represent the best outcomes on the QLQ-C30 functioning scales and the worst outcomes on the QLQ-C30 and QLQ-H&N35 symptom scales. A higher score of symptom scale represents a more pronounced problem, which gives a poorer HRQoL (17,18). The Serbian versions were provided by the EORTC Group.

- Oral Health Impact Profile - OHIP 14.

The OHIP-14 instrument has 14 items about oral health status (19). The total score is the sum of all answered items and can range from 0 to 56. The higher the score, the worse the impact on oral health (19). The Serbian version was previously developed and validated for HNC patients (20).

- European Health Interview Survey - EHIS.

Background data (demographic and socio-economic), health status (including comorbidities) and health determinants (life style) were assessed using the EHIS (21). The Serbian version of the EHIS was provided by the Institute of Public Health of Serbia “Dr Milan Jovanović Batut”. The EHIS survey incorporated several validated instruments, including: Oslo Social Support Scale (OSSS-3), Personal Health Questionnaire Depression Scale (PHQ-8), and basic and instrumental ADLs (21).

The OSSS-3 has three items measuring levels of social support (22). The total score is the sum of the questions answered and can be in a range of 3 to 14, where a higher score indicates a higher level of social support. The total score is divided into three groups of support level: low (3-8 score), moderate (9-11 score) and high (12-14 score).

The Personal Health Questionnaire (PHQ-8) has 8 items related to depressive symptoms (23). The total score represents the sum of all the questions answered and it can range from 0 to 24. A score of 10 represents clinically relevant depressive symptoms.

The instruments for ADLs and IADLs measure basic and instrumental physical activities (24). All questions have answers rated on a 3-point Likert scale (from 0 = not at all, to 3 = no difficulty). The total score for ADLs is between 0 and 15, and for IADLs between 0 and 21. A lower score indicates a higher difficulty in performing basic and instrumental activities of a daily life.

- Data analysis

Regression analyses (i.e., forward stepwise method) were used to analyze HRQoL predictors considering the previous studies (4,25). The dependent variable was the score on the QLQ-C30 questionnaire, while independent variables were all demographic and clinical parameters and individual symptoms from the QLQ-C30 and H&N35 questionnaires. The number of respondents was determined according to the G * Power program (26). At least 220 subjects would be required to apply linear regression in the analysis of up to 20 predictor variables for α = 0.05 and power 1-β = 0.95. Since all of the participants did not answer all the questions, the sample size varied for different results (ADLs). A value of *p* < 0.05 is considered statistically significant.

## Results

Demographic and socio-economic status, health status (including comorbidities) and health determinants of participants, as well as clinical characteristics are given in the Table 1.

- Influence of HRQoL predictors on HNC patients

Nine regression analyzes were conducted to test the dependent variables of the six EORTC QLQ-C30 scales, OHIP scale, and the ADLs & IADLs scales, to determine predictors of HRQoL of HNC patients (Tables 2, Table 3).

**Figure 1:**
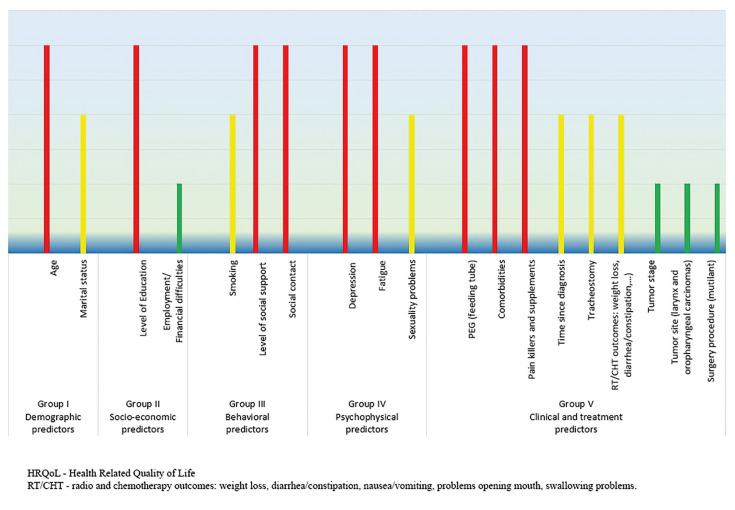
HRQoL Predictors’ Impact on HNC Patients.

In total 19 predictors had a significant, a moderate and a small impact on HRQoL, and these predictors are classified into five groups (Fig. 1).

Of all identified predictor variables, PEG tube was statistically associated with lower scores in 8 of 9 domains included in the HNC HRQoL (*p*<0.01). Comorbidity was a predictor of HRQoL that affected global, role, cognitive and social functioning and should be observed as an independent variable in QoL research (*p*<0.05). Age influenced 4 of the 6 QLQ-C30 functional scales as well as the OHIP score and ADLs (*p*<0.05), while education level significantly affected most aspects of QoL including 4 of the 6 QLQ-C30 functional scales, the OHIP score, and the ADLs/IADLs scales (*p*<0.05). Marital status influenced on physical functioning, global score and ADLs (*p*<0.01).

Employment/financial difficulties was a predictor of physical and social functioning (*p*<0.01), while analyzed separately, had impact on OHIP score, treatment type, tumor site and stage and time since diagnosis (*p* = 0.01). The level of social support and social contact (p≤0.01; *p*<0.05), as well as fatigue (p≤0.01; *p*<0.05), affected 5, i.e., 4 functional scales and the basic and instrumental ADLs. Pain and the use of painkillers and supplements had an impact on 5 of the 6 functional scales and on basic ADLs (*p*<0.01). Depression was statistically associated with lower scores in 4 of the 6 functional scales as well as with ADLs (*p*<0.05), and sexual problems had a similar impact, influencing 4 of the 6 functional scales and the OHIP score (*p*<0.05). Smoking, the presence of a tracheostomy and the time since diagnosis were predictors with moderate impact on QoL (*p*<0.05). Those who completed the questionnaires more than a year after their diagnosis had statistically higher QoL scores globally and for all five scales measuring functionality, compared to the other two groups (Bonferroni post-hoc analysis; *p*<0.01). Also, there is a significant statistical difference in the values of OHIP-14 scores in relation to the time since the diagnosis. Respondents surveyed between 1 and 12 months after diagnosis (when they are most likely to be receiving therapy) have the highest scores on the OHIP questionnaire (*p*<0.01).

Certain types of therapy, primarily a multimodal approach to the treatment that included both RT and CHT, showed to be moderate predictor of QoL with the greatest impact on H&N35 symptom scales: weight loss, diarrhea/constipation, nausea/vomiting, mouth opening problems and swallowing problems (*p*<0.01). In addition to this, regarding tumor site, results showed that patients with laryngeal cancer and oropharyngeal cancers, compared with patients with tumors of other site, had significantly higher scores of symptoms of fatigue, nausea and vomiting, insomnia, loss of appetite and constipation (*p*<0.01). Patients with larynx cancer and oropharyngeal cancers (oral cavity and pharynx) and patients who underwent RT/CHT in addition to surgical treatment had higher OHIP scores, than patients with other cancer site and patients who underwent only surgical treatment (*p*<0.01). Also, groups with oropharyngeal cancers and larynx cancer had significantly lower scores of global, physical, emotional and social functioning compared to patients who had tumor of another site (*p*<0.01). However, we observed that cognitive and Role functioning scores were significantly lower only in patients with oropharyngeal cancers, Table 4. Tumor stage (*p*<0.05) and performed surgery with negative anatomic and functional outcomes (*p*<0.01) were significant in a group diagnosed with tumor in the stage III/IV, with small impact on HRQoL, see Table 3.

**Table 2:**
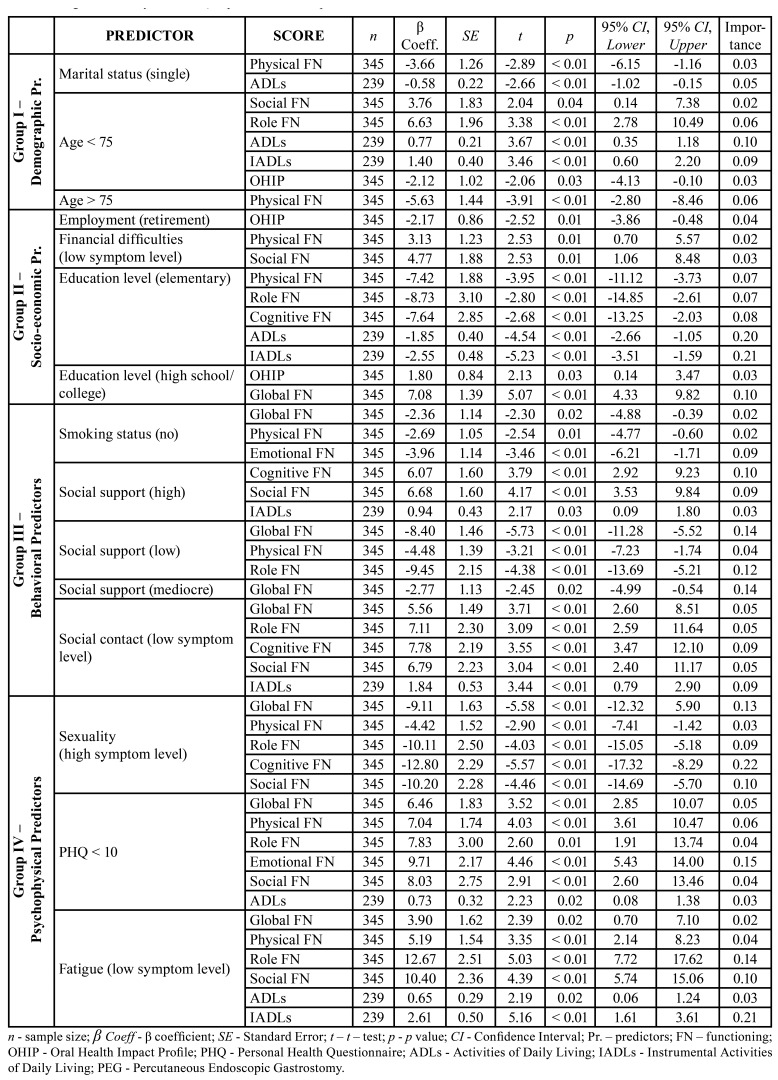
Regression analysis of HRQoL predictors - Groups I - IV.

**Table 3:**
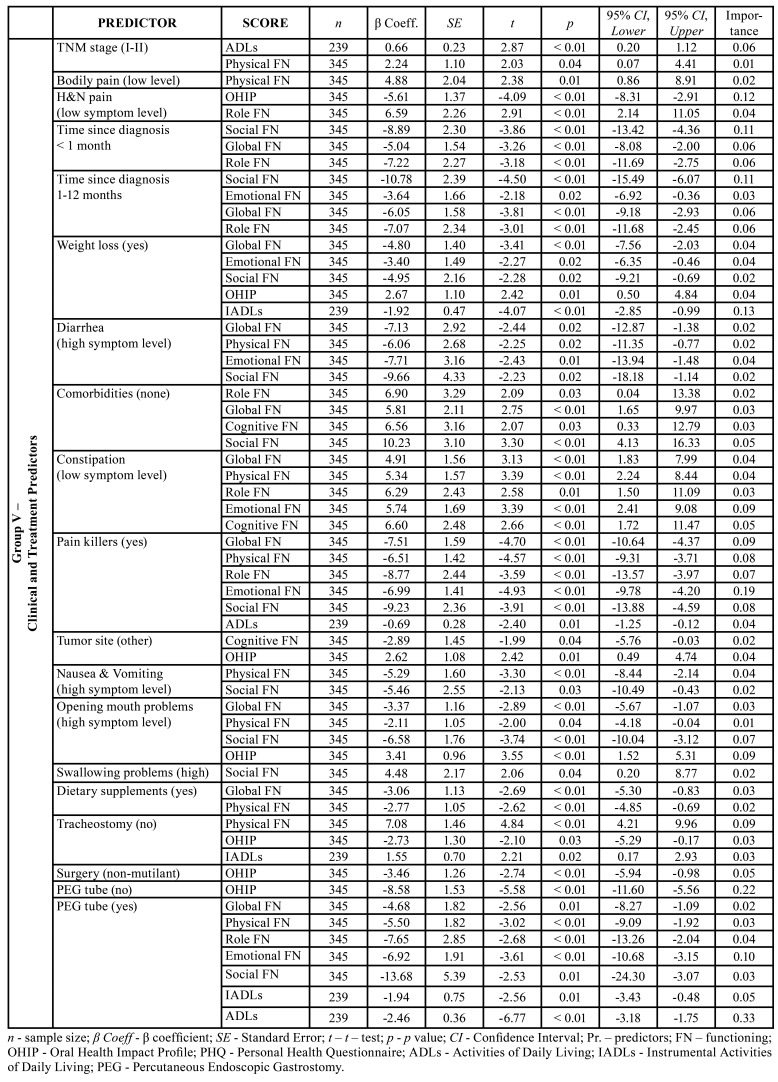
Regression analysis of HRQoL predictors - Group V.

**Table 4:**
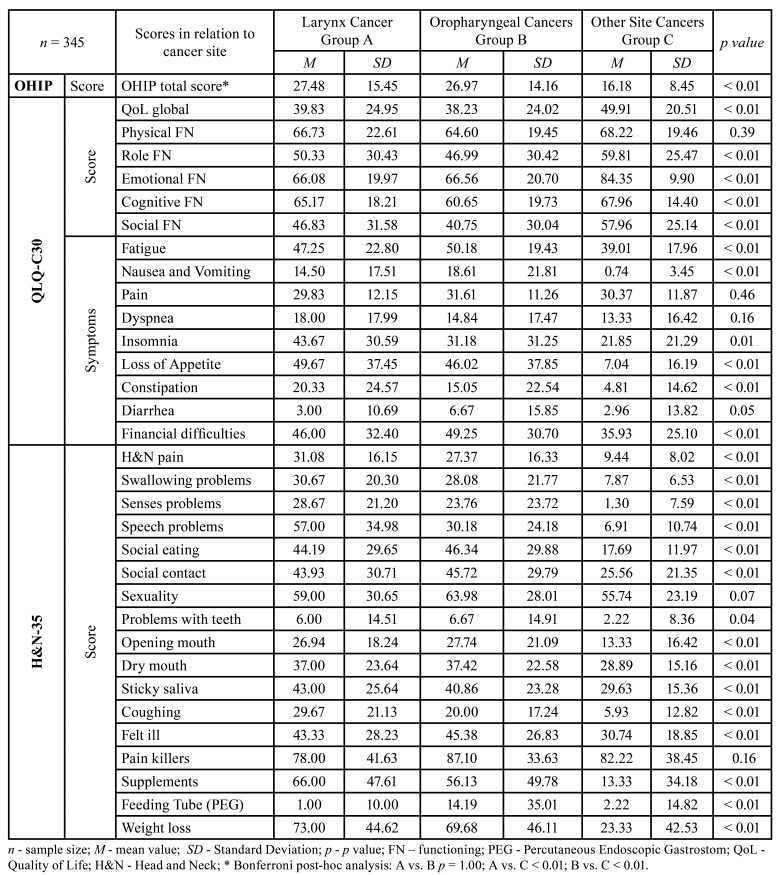
Questionnaire scores in relation to cancer site.

## Discussion

The demographic and socioeconomic aspect of the participants of our study matches with the epidemiological profile of the HNC found in literature, i.e. an older men, with completed elementary and/or high school, who were in some kind of partnership or single and exposed at least to one risk factor, such as smoking and/or alcohol, with poor economic situation (9,10). Because multiple QoL scales are affected and patients are compelled to make lasting changes in their eating, swallowing, and communication habits, differences in QoL are expected among the HNC patients’ groups depending on their age, marital, and educational background and employment status (4,8).

In our study, older age, single marital status, and low level of education, were shown to be significant predictors of lower global QoL and poorer physical functioning, particularly in the group with oropharyngeal carcinomas. The results also showed the lowest score for ADLs for males over the age of 75, who were single and had only elementary school education. Level of education has significantly affected global, physical, role and cognitive functioning, and the OHIP score and ADLs and IADLs, making it the most dominant independent socio-economic predictor. Single marital status primarily negatively affected physical functioning, global score and ADLs which supports a previously determined fact that the QoL is better in patients with partner, and a sTable relationship is an important modifying factor of psychological distress, depression and better utility (13,27).

A strong social support was experienced by 34.2% of our respondents, while 40.3% experienced a mediocre support and 25.5% claimed a low social support. The results and percentage of the social support assessment in the HNC patients may vary from study to study, but all authors agree that the social support has a strong impact on the QoL in these patients, both during the treatment and after it (12).

Although smoking and alcohol consumption are the main cause of the HNC in about 75% of cases (2), as many as 89.6% of our study participants reported consuming alcohol up to twice a month, and 10.4% a couple of times a week. Among all participants, 28.1% were smoking regularly. Prolonged alcohol and tobacco consumption after diagnosis and during the treatment increases the risks significantly, not only from premature death, but also from causing the secondary tumor in more than one third of the HNC patients (2). Prior review studies and HNC guidelines showed that smoking and alcohol consumption are predictors of a prolonged need for nutrition by gastrostomy, which labels these habits as significant predictors of the global QoL (2).

Based on our study, it is apparent that the most frequent QoL predictor is PEG tube (gastrostomy) and it is more influential than presence of the tracheostomy. Our patients with gastrostomy had a significantly higher OHIP score, and lowered global, physical, role, emotional and social functioning, with reduced ADLs and IADLs at the same time. Similar to these, patients with placed tracheostomy reported impaired physical functioning, poorer IADLs and higher OHIP score. In addition to that, 94.6% of our patients included suffered from one or more comorbidities and hence, they had lower scores in four out of six scales - global, role, cognitive and social functioning. Previous study of Boje *et al*. found that the older the age the more frequent the comorbidities, so that the largest number of chronic diseases was reached around the age of 70 (28). This is in line with our findings of the average age of 69.65 years old. This deterioration of HRQoL on QLQ-C30 scales caused by presence of PEG tube, tracheostomy, comorbidities (associated with older age) continues as symptoms of fatigue, depression and sexuality problems are more pronounced, which is particularly observed in patients with oropharyngeal and laryngeal carcinomas, compared to patients with other cancer site; based on the clinical interview during the survey, 37.1% of patients had a PHQ score over ten, i.e. in the clinical range of significant symptoms of depression. Among the general symptoms, 52% of our patients claimed a low level, and 48% a high level of Fatigue. A low level of Sexuality was declared by 76.8%, and a high level by 23.2% patients.

Also, our study showed that the most pronounced symptoms as negative outcomes of RT/CHT were loss of appetite (observed in 41.2% of respondents), difficulties with opening the mouth (63.4% of respondents), swallowing problems (41.6% of respondents), dry mouth (20.6%), constipation (present in 9.9% of respondents) and diarrhea (2.3% of respondents). Weight loss during treatment was reported by 58.6% of respondents. At the same time, and in our results, the lower score of the respondents for physical functioning, Bansal *et al*. explained by a causal relationship between deterioration in physical functioning and increased symptoms such as fatigue, pain, depression, and loss of appetite (29). In our study, patients with a high level of fatigue, lower level of education and social support, who had PEG, tracheostomy and experienced a weight loss, showed worse ADLs and IADLs scores, which correlates with the results of Blanco *et al*. who showed that the more pronounced the symptoms of pain, fatigue and weight loss, the more pronounced the decrease on the functional scale, with the loss of physical, social and emotional functions and role functioning (30). The analysis of our study shows that the most common limitations related to basic ADLs are the inability to perform personal hygiene and going for a walk, while for instrumental ADLs are the inability to do housework, shopping and use of public transport, which is also the conclusion of Neo at al. (16). In general, instrumental ADLs requiring physical functions were more commonly affected than those requiring cognitive functions.

In our study, H&N pain proved to be a significant predictor variable, particularly in groups of patients with laryngeal cancer and oropharyngeal cancers. It can be said that in this case, H&N pain is a predictor of cancer localization, since these two groups of patients had significantly higher scores on the scales of both total QoL and H&N-35 modulus. Also, these two groups of patients on the scales of the H&N35 module - Painkillers and Supplements, had significantly higher scores than patients with cancer of other localization. Likewise, on the scale of general QoL symptoms, a prominent level of pain during treatment was reported by 4.3% of patients, and a low level by 95.7%. Yet, 38.8% of them claimed for the presence of elevated levels of pain in the head and neck, and 61.2% for low levels. During treatment process, 83.2% of the patients in our study used painkillers, while 16.8% did not use them. Also, 47.8% of respondents used various supplements. These findings confirm the previously established fact that high standard deviation of Pain scale from the mean, suggests a large fickleness in its perception (9) and that it is necessary to ensure proper nutrition of patients regardless of the increased use of pain killers and dietary supplements.

Regarding the influence of time passed since a diagnosis, our study showed a deterioration in HRQoL in the domain of global and role functioning, as well as emotional and social functioning, especially during the first month of initiated treatment. This deterioration continues for up to 12 months from diagnosis and ongoing treatment (RT/CHT), indicating both a dominant influence of treatment modality and the time since diagnosis. Also, those who completed the questionnaires more than 12 months after diagnosis, had statistically higher global QoL scores, as well as for all five scales measuring functionality and the lowest OHIP scores (Bonferroni post-hoc analysis; *p*<0.01).

The obtained results reflect previous researches, in which it has been proven that over time there is a gradual improvement in the QoL of patients with HNC and that patients in whom 12 or more months have passed since diagnosis had better scores related to QoL predictors (1,4,6,14). This can be explained by the fact that over time, the negative consequences of illness and its treatment are really mitigated, but also with the gradual adjustment and acceptance of the recent changes in health status by the patients themselves. Also, it was shown that the patients who experienced stronger social support either by their family members, friends, colleagues or group therapy could cope with the treatment consequences more easily, have recovered more quickly and finally accepted the new life circumstances they found themselves in (12).

Tumor site and stage undoubtedly have not only strong prognostic significance on the course of the disease but also on the functioning of the patient (4,5). It must be noted that in our study cancer site, stage and the performed surgery proved to be low significant predictors of QoL, primarily with an impact on cognitive and physical functioning and daily life activities. We found that in patients who underwent mutilant (disFiguring) surgery (neck dissection, total laryngectomy, partial mandibulectomy/maxillectomy) as well as in those who underwent postoperative RT/CHT the scores on the ADLs and IADLs scales were significantly lower comparing with patients with a cancer of other site and those in whom only surgical treatment was performed. Also, the results of few studies showed that patients with advanced tumor stage (stage III and IV) have a far poorer QoL compared to patients with tumor stage I and II, both at the time of diagnosis and after one year of follow-up (5,6,14), which is also confirmed by the results of our study.

The limitations of our study, are the difficulties in obtaining a satisfactory sample. As part of a larger intervention venture to assess HRQoL, a higher decrement should be expected in a number of scales, observed within various predictors. A likely reason for such results is the large number of different cancer localizations and different surgical procedures performed and in unequal numbers in all participants, which consequently, despite the statistical attempt to homogenize the sample, inevitably leads to its dispersion. Consequently, QoL decrements that could be expected in population with different cancer localization and stages may be related to therapy and number of therapeutic modalities rather than tumor site and stage, making them less significant predictors of QoL in multivariate analyzes as our study is.

## Conclusions

Study identified nineteen predictors that had significant, moderate and small impact on the HRQoL of patients with HNCs. Some of the predictors, like levels of social support and social contact, depression, and comorbidities could be targets for innervations to improve HRQoL. Future studies should address greater emphasis on the development of strategies for improving the QoL and drivers of changes in public health programs, based on already established facts.
